# Long‐acting injectable ART to advance health equity: a descriptive analysis of US clinic perspectives on barriers, needed support and programme goals for implementation from applications to the ALAI UP Project

**DOI:** 10.1002/jia2.26282

**Published:** 2024-07-05

**Authors:** Nadia Nguyen, Benjamin Lane, Sarit A. Golub, Cody Chastain, Jason Zucker, Katherine King, Marvell Terry, Jennifer Burdge, Caroline Carnevale, Anahit Muscarella, Delivette Castor, Bryan Kutner, Kathrine Meyers

**Affiliations:** ^1^ Aaron Diamond AIDS Research Center Columbia University Irving Medical Center New York City New York USA; ^2^ Hunter College of the City University of New York New York City New York USA; ^3^ Division of Infectious Diseases Department of Medicine Vanderbilt University Medical Center Nashville Tennessee USA; ^4^ Division of Infectious Diseases Department of Medicine Columbia University Irving Medical Center New York City New York USA; ^5^ New York City Department of Mental Health and Hygiene New York City New York USA; ^6^ New York Presbyterian Hospital New York City New York USA; ^7^ Albert Einstein College of Medicine Bronx New York USA

**Keywords:** Consolidated Framework for Implementation Research, equity, health disparities, implementation science, injectable cabotegravir rilpivirine, long‐acting injectable HIV treatment

## Abstract

**Introduction:**

Approval of the first long‐acting injectable antiretroviral therapy (LAI ART) medication heralded a new era of HIV treatment. However, the years since approval have been marked by implementation challenges. The “Accelerating Implementation of Multilevel Strategies to Advance Long‐Acting Injectable for Underserved Populations (ALAI UP Project)” aims to accelerate the systematic and equitable delivery of LAI ART.

**Methods:**

We coded and analysed implementation barriers according to the Consolidated Framework for Implementation Research (CFIR) domains, desired resources and programme goals from questionnaire short‐answer responses by clinics across the United States responding to ALAI UP's solicitation to participate in the project between November 2022 and January 2023.

**Results:**

Thirty‐eight clinics responded to ALAI UP's solicitation. The characteristics of LAI ART as an innovation (cost, complexity of procurement, dosing interval, limited eligibility) precipitated and interacted with barriers in other CFIR domains. Barriers included obtaining coverage for the cost of medication (27/38 clinics) (outer setting); need for new workflows and staffing (12/38) and/or systems to support injection scheduling/coordination (16/38), transportation and expanded clinic hours (13/38) (inner setting); and patient (10/38) and provider (7/38) education (individuals). To support implementation, applicants sought: technical assistance to develop protocols and workflows (18/38), specifically strategies to address payor challenges (8/38); additional staff for care coordination and benefits navigation (17/38); opportunities to share experiences with other implementing clinics (12/38); patient‐facing materials to educate and increase demand (7/38); and support engaging communities (6/38). Clinics’ LAI ART programme goals varied. Most prioritized delivering LAI ART to their most marginalized patients struggling to achieve viral suppression on oral therapy, despite awareness that current US Food and Drug Administration approval is only for virally suppressed patients. The goal for LAI ART reach after 1 year of implementation ranged from ≤10% of patients with HIV on LAI ART (17/38) to ≥50% of patients (2/38).

**Conclusions:**

Diverse clinic types are interested in offering LAI ART and most aspire to use LAI ART to support their most vulnerable patients sustain viral suppression. Dedicated resources centred on equity and relevant to context and population are needed to support implementation. Otherwise, the introduction of LAI ART risks exacerbating, not ameliorating, health disparities.

## 1 INTRODUCTION

Long‐acting injectable antiretroviral therapy (LAI ART) can dramatically transform HIV care and reduce HIV‐related health inequities in achieving and maintaining viral suppression [1, 2, 3]. However, in the first 2 years since the US Food and Drug Administration (FDA; a US institution) approval of the first LAI ART injectable, cabotegravir rilpivirine (iCAB/RPV), implementation and uptake of iCAB/RPV has been slow, with one industry analysis reporting that in the first year post‐approval, only 1–2% of patients had switched to iCAB/RPV [4]. These findings are echoed in two early implementation studies at Ryan White HIV/AIDS Program‐funded clinics in San Diego, California and Atlanta, Georgia, which reported that only a quarter to a half of patients who expressed interest in iCAB/RPV were able to initiate iCAB/RPV within 12 months of initial interest [5, 6]. For LAI ART to accelerate progress towards Ending the Epidemic goals, clinics urgently need support to integrate LAI ART into their clinical practice and equitably deliver LAI ART at scale.

Historically, pharmacological innovations in HIV have exacerbated disparities. In the 10 years following the introduction of highly active antiretroviral therapy in 1996, disparities in HIV mortality between Black versus White populations increased almost eight‐fold [7]. The same pattern has repeated with pre‐exposure prophylaxis (PrEP), with differential uptake of PrEP between White gay and bisexual men who have sex with men (MSM) versus Black MSM, contributing to a decrease in HIV incidence by 20% in White MSM, but only 4% in Black MSM [8].

For LAI ART to upend this pattern of exacerbated health disparities, the delivery of LAI ART must be prioritized for populations who are currently not being well‐served by available oral treatments, including people who are out of care and people who are not reliably virally suppressed due to suboptimal adherence [1, 9, 10, 11]. To date, there is limited real‐world evidence explicating and articulating strategies on how to overcome barriers to equitable delivery of LAI ART and how to best support clinics in reaching patient populations who could benefit most from this biomedical innovation [3, 12, 13, 14, 15].

In September 2022, we launched Advancing Long‐Acting Injectables for Underserved Populations (The ALAI UP Project), a Special Project of National Significance funded by the Department of Health and Human Services Minority HIV/AIDS Fund and the Health Resources & Services Administration (two US institutions) designed to support clinics across the United States to develop injectable HIV treatment programmes (starting with iCAB/RPV) that prioritize the needs of underserved populations. Through a collaboration between Columbia University Irving Medical Center, Hunter College of the City University of New York, the NYC Department of Health and Mental Hygiene, and the Southeast AIDS Education and Training Center at Vanderbilt University Medical Center, over the next 3 years, eight selected clinical sites will receive sample protocols to support the equitable delivery of iCAB/RPV, technical assistance to tailor and implement the protocols, monitoring and evaluation support to inform continuous quality improvement and help engaging communities and peer support from other implementing clinics.

We report our early learnings from the clinical site application and selection process of the ALAI UP Project, including site‐reported barriers and facilitators to equitable implementation, requested supports, implementation goals, and how we used this information to design a multicomponent package of implementation supports that has the overarching and explicit goal of advancing equity in health outcomes through strategic delivery of LAI ART to underserved populations.

## 2 METHODS

### 2.1 Study samples and procedures

The ALAI UP Project published a request for applications on our website (https://www.alai‐up.org) in November 2022. We advertised the request for applications via outreach to state and local health departments, HIV advocacy groups, public health and academic listservs, and our professional networks and hosted an informational webinar for interested clinical sites in December 2022. To be eligible, clinical sites needed to: (1) be located in any state in the United States or Territory, and (2) provide HIV treatment to at least 25 people. Additionally, sites needed to designate a Project Champion with some decision‐making authority and identify a person who would support data reporting. Interested clinics submitted applications on REDCap in January 2023. The ALAI UP application included a Clinical Site Questionnaire consisting of multiple choice and short answer questions regarding patient and clinic characteristics, stage of iCAB/RPV implementation, future iCAB/RPV programme goals, capacity and commitment to deliver iCAB/RPV equitably, barriers and facilitators to iCAB/RPV implementation, and supports needed. Selected sites were notified in mid‐February 2023 and were enrolled into ALAI UP in March 2023. We summarize findings from a secondary analysis of the Clinical Site Questionnaire. All study procedures were reviewed and approved by the Columbia University Irving Medical Center's Institutional Review Board.

### 2.2 Measures

The following measures were captured in the Clinical Site Questionnaire (see Additional File S1), which were submitted by the clinic as part of their application to participate in ALAI UP.

#### 2.2.1 Social determinants of health

Clinics reported the proportion of their patients (none, a few, about half, most, all) with food insecurity, housing instability, primary language other than English, financial strain and lack of transportation/access to public transportation to attend clinic visits.

#### 2.2.2 Size of current iCAB/RPV programme and goal for iCAB/RPV reach

Clinics reported on the date of their first patient initiated on iCAB/RPV and the number of patients currently on iCAB/RPV. To understand clinics’ goals for iCAB/RPV programme growth and reach, clinics reported the proportion of patients with HIV they hoped to initiate on iCAB/RPV after 12 months of implementation (reach) with ALAI UP support.

#### 2.2.3 Barriers to iCAB/RPV implementation and needed resources

Clinics reported their top three anticipated or experienced barriers to implementing iCAB/RPV as well as resources needed to support equitable implementation of iCAB/RPV.

#### 2.2.4 Clinic potential to use LAI ART as a tool to advance health equity

Clinics reported on their mission statement, wrap around services, and gave examples of how lessons learned from community engagement and addressing social or structural barriers to HIV care informs how they deliver/will deliver iCAB/RPV. Clinics also reported the populations they will prioritize for iCAB/RPV delivery.

### 2.3 Data analysis

#### 2.3.1 Quantitative analysis

Categorical variables (clinic characteristics, implementation goals, priority populations) were exported from REDCap into Microsoft Excel and summarized using frequency and proportions.

#### 2.3.2 Qualitative analysis

Short answer, qualitative responses related to clinic barriers and facilitators to iCAB/RPV implementation were analysed by a team of three researchers using Framework Analysis and coded according to the following four relevant domains from the Consolidated Framework for Implementation Research (CFIR) (characteristics of iCAB/RPV as an innovation, outer setting, inner setting and characteristics of individuals) [16, 17]. ALAI Up defines equitable implementation of LAI ARTs as: “The prioritization of resources to address barriers that underserved populations face in accessing long‐acting injectable antiretroviral therapy (LAI ART) so that all people with HIV (PWH) can access the HIV treatment modality that most effectively allows them to thrive and reach the highest attainable standard of health.” Clinics’ potential to use LAI ART as a tool to advance health equity was examined by coding specifically for evidence of human equality‐centredness and recipient‐centredness (sub‐constructs in the culture construct within the inner setting domain). The coders generated code reports for each CFIR domain, identified key quotes, and iteratively discussed key themes with the larger team and subsequently recoded text when appropriate.

## 3 RESULTS

### 3.1 Clinic characteristics

Thirty‐eight clinics from across the United States responded to the ALAI UP application solicitation (Table 1). Clinic type included academic medical centres (AMCs), hospitals, departments of health, AIDS service organizations (ASOs), community‐based organizations, federally qualified health centres, community health centres and primary care clinics. AMCs, ASOs and primary care clinics were the most common applicant clinic types (together making up 24 clinic applicants, 63%), while departments of health were the least common (*n* = 3, 8%).

**Table 1:** Characteristics of clinical site applicants to ALAI UP

	*N* (%) of clinics
Clinic type	
Academic medical centre	8 (21%)
Hospital	5 (13%)
Department of health	3 (8%)
AIDS service/community‐based organization	8 (21%)
Federally qualified health centre	6 (16%)
Community health centre/primary care	8 (21%)
Geographic region	
West	7 (18%)
Southwest	9 (24%)
Midwest	4 (11%)
Southeast	8 (21%)
Northeast	10 (26%)
≥50% of patients with social determinant of health	
Food insecurity	28 (74%)
Housing instability	24 (63%)
Primary language other than English	20 (53%)
Financial strain	38 (100%)
Lack of transportation/access to public transportation	27 (71%)
Number of patients on iCAB/RPV	
Has not started implementation of iCAB/RPV	8 (21%)
Initiated <25 patients on iCAB/RPV	23 (61%)
Initiated ≥25 patients on iCAB/RPV	7 (18%)
Number of patients with HIV in care^a^	
<100	5 (14%)
100 to <300	7 (19%)
300 to <1000	10 (28%)
1000 to <2000	8 (22%)
≥2000	6 (17%)
iCAB/RPV reach goal after 1 year of implementation^b^	
≤10% of patients	17 (46%)
11–25% of patients	15 (41%)
>25% of patients	5 (13%)
Priority population for iCAB/RPV^c^	
Black/Latinx	13 (34%)
LGBTQ, gender non‐conforming	11 (29%)
Youth	8 (21%)
Older adults	2 (5%)
Women	6 (16%)
Unstably housed	9 (24%)
Substance use challenges	6 (16%)
Mental health challenges	3 (8%)
Privacy concerns	6 (16%)
Pill fatigue/aversion	10 (26%)
Patients with adherence challenges	8 (21%)
“Ideal” patients (oral medication and visit adherent)	4 (11%)
Newly diagnosed	3 (8%)
Clinics with the following staff type involved in HIV‐related care	
Nurse practitioner (NP) or doctor (MD)	38 (100%)
Registered nurse (RN)	32 (84%)
Social worker	33 (87%)
Benefits coordinator	34 (89%)
Peer navigator	23 (61%)

Abbreviations: ALAI UP Project, Accelerating Implementation of Multilevel Strategies to Advance Long‐Acting Injectable for Underserved Populations; iCAB/RPV, injectable cabotegravir/rilpivirine.

^a^ Number of patients with HIV who received care defined as all patients with HIV who had at least one CD4 or viral load test in 2021 at clinic.

^b^ Clinic self‐reported goal for proportion of patients with HIV on iCAB/RPV after 1 year of implementation with ALAI UP support.

^c^ Reported priority populations were not mutually exclusive; clinics could report more than one priority population.

### 3.2 Social determinants of health

Clinics served patients with multiple social determinants of health needs, with a high percentage of clinics reporting that over half their patients experienced: food insecurity (*n* = 28, 74%), housing insecurity (*n* = 24, 63%), financial strain (*n* = 38, 100%) and lack of transportation (*n* = 27, 71%). In addition, 53% (*n* = 20) of sites reported that more than half of their patients had a primary language other than English.

### 3.3 Size of current iCAB/RPV programme

Clinics with iCAB/RPV programmes of various sizes submitted applications, with the most applications from clinics that had fewer than 25 patients on iCAB/RPV (*n* = 23, 61%), followed by clinics that had no iCAB/RPV patients (*n* = 8, 21%), and finally clinics that had 25 or more patients on iCAB/RPV (*n* = 7, 18%). Among the 30 clinics with at least one patient on iCAB/RPV, only one clinic began delivering iCAB/RPV in 2020, nine clinics began in 2021, 19 in 2022 and one in 2023.

### 3.4 Goal for iCAB/RPV reach

**Figure 1:**
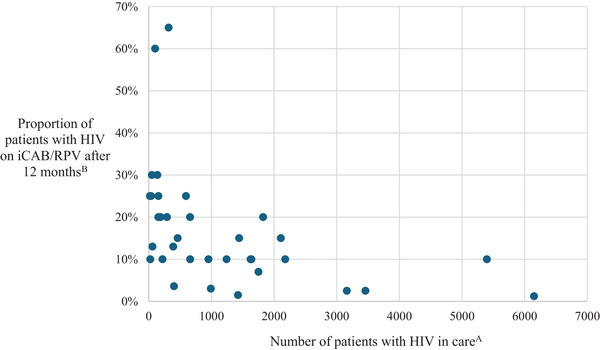
**Goal for injectable cabotegravir/rilpivirine (iCAB/RPV) reach among patients with HIV after 12 months of implementation by number of patients with HIV served at clinic**. (A) Number of patients with HIV who received care defined as all patients with HIV who had at least one CD4 or viral load test in 2021 at clinic. (B) Clinic self‐reported goal for proportion of patients with HIV on injectable cabotegravir/rilpivirine (iCAB/RPV) after 1 year of implementation with ALAI UP (Accelerating Implementation of Multilevel Strategies to Advance Long‐Acting Injectable for Underserved Populations) support.

Clinics varied widely in their projected reach for their iCAB/RPV programmes after 12 months of implementation, ranging from 1% of their HIV patient population on iCAB/RPV to 65%. The majority (*n* = 22, 57%) set goals for 15% or fewer of their patients on ART being on iCAB/RPV after 1 year, while a third (*n* = 12, 32%) set goals between 16% and 30% (Figure 1). Only two clinics set goals for more than 50% of patients on iCAB/RPV: one is a small ASO that serves approximately 100 patients with HIV, while the other is a hospital‐affiliated clinic serving just over 300 patients with HIV. Some clinics were reluctant to set goals because, as one DOH applicant noted, “*long‐acting injectable therapy is not a superior treatment to oral regimens and is a patient‐specific decision [therefore] our goal is to be able to meet interest and offer injectable therapy to all patients who meet clinical criteria for switching and want to switch.”* Goals for reach did not vary by clinic type with a median goal of 10–13% across all clinic types. However, overall, we found that clinics serving fewer patients on ART (<100) believed that iCAB/RPV could be a bigger part of their ART programme, while larger programmes with more than 3000 patients on ART set iCAB/RPV goals at 1–2% of patients on iCAB/RPV.

### 3.5 Barriers to implementation

**Figure 2:**
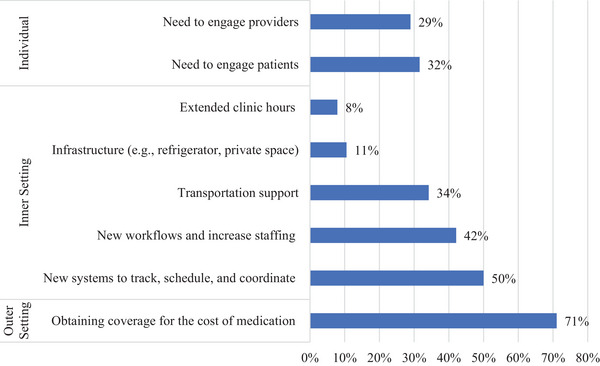
**Proportion of clinics reporting Consolidated Framework for Implementation Research (CFIR) domains**.

Clinics reported many common barriers to implementation (Figure 2); however, the specific characteristics of iCAB/RPV itself as an innovation—specifically its high cost and complexity and limited adaptability—precipitated and interacted with barriers across all other CFIR domains. For example, the high cost of iCAB/RPV relative to oral ART (innovation cost) necessitates that most patients must obtain coverage for the cost of the medication (either through insurance or medication assistance programmes), with clinics reporting that navigating and obtaining coverage for the cost of medication is the most salient barrier to iCAB/RPV implementation (outer setting financing). Similarly, the complexity of procurement from specialty pharmacies, need to maintain cold chain and refrigerated storage, and the dosing interval (innovation complexity) necessitate robust clinic systems for tracking and coordinating injection visits and medication shipments, and new workflows, task shifting and additional staffing to support this more labour‐intensive treatment (inner setting infrastructure, culture and compatibility with existing workflows). Finally, the narrow eligibility according to the current iCAB/RPV label and available clinical trial data, in addition to the strict dosing window (innovation adaptability), creates a tension among providers and clinic administrators who may want to provide iCAB/RPV to patients who can benefit most from this new treatment (including patients who are not adherent to oral ART), but have concern about a patient's ability to attend injection visits on time and potential for drug resistance (inner setting and individuals domain) (Table 2).

**Table 2:** Barriers to injectable cabotegravir/rilpivirine (iCAB/RPV) implementation grouped by Consolidated Framework for Implementation Research (CFIR) domain

CFIR domain	Barriers to iCAB/RPV implementation
**Outer setting**	Time spent navigating insurance challenges: “*Medication access is a significant barrier to expanding our Cabenuva workflow. Cabotegravir /rilpivirine is not available through the ADAP [AIDS Drug Assistance Program] formulary or the Ryan White Part A Assistance Program formulary. The prior authorization process for insured patients eligible to participate in our current Cabenuva workflow is a time‐consuming [and] complex web of administrative coordination with insurers, health system benefits eligibility, and patients*.” (Academic Medical Centre)
	Increased administrative burden: “*Each insurance plan has various administrative rules which are complex to navigate. Almost all require prior authorization and again as a resource limited provider the increased administrative burden increases our operational cost which ultimately limits the care we can provide. Moreover, the prior authorization process is not well understood at the insurer level as many plans are still asking for inappropriate information, e.g., request for coverage denied because patient has not failed other therapies. Providers are spending a great deal of time doing peer to peer reviews to educate insurers that they are not following FDA [US. Food and Drug Administration] prescribing guidance*.” (AIDS Service Organization)
	Medical versus pharmacy benefit: “*Time and resources to determine insurance coverage (medical or pharmacy benefit) and the time required to complete prior authorization for the medication*” are a significant barrier to iCAB/RPV scale up. (Academic Medical Centre)
**Inner setting**	Need for care coordination systems: “*Communication regarding follow‐ups with patients will be critical, but can be difficult, especially for our priority populations. It is essential for patients to come in for injections on schedule, therefore we will need to improve/increase patient follow up and communication via phone, text, MyChart [a secure service that allows patients to access their health information and communicate with their care team], etc., with fall‐back plans to prescribe and deliver oral medications for patients who delay or miss an injection*.” (Academic Medical Centre)
	New workflows and increased staffing: “*Like other healthcare delivery settings, we are increasingly encountering higher patient volumes and difficulty increasing staff to match the demand. The provision of long‐acting cabotegravir/rilpivirine is time intensive and includes patient education, patient tracking, insurance approval, medication administration, and troubleshooting missed or delayed doses*.” (Department of Health)
	New storage space and fridge: “*Currently, we can only accommodate roughly three or four patients’ injections per day. With […] funding for medication fridges, we anticipate being able to replicate and or improve upon our original model and expand to our outreach clinics which would alleviate staffing constraints and allow patients to receive the medication within their preferred clinic location, close to home*.” (Academic Medical Centre)
	Extended hours: “*Currently, we can only administer long‐acting cabotegravir/rilpivirine during normal working hours, which prohibits patients with inflexible work schedules as they may not be able to present to clinic every 8 weeks for the injections*.” (Department of Health)
**Individual—innovation recipients (patients)**	Lack of awareness among patients: “*We have experienced a general lack of knowledge and awareness of long‐acting HIV therapies among our clinic patients. Prior to deciding to respond to this funding opportunity, we informally surveyed a small subset of patients, of which >50% had never heard of long‐acting cabotegravir/rilpivirine. Of these patients, >60% would consider switching to long‐acting cabotegravir/rilpivirine upon further discussion with their clinical provider*.” (AIDS Service Organization)
	Patient hesitancy about new treatment: “*We anticipate hesitancy from the community for the injectable form of the medication vs oral. Lingering misinformation surrounding COVID‐19 vaccination has led to a trickle‐down effect where patients have a generalized mistrust of all injectables, including antibiotics used for the treatment of sexually transmitted infections (STIs). It is likely this mistrust would extend to long acting cabotegravir/rilpivirine, at least initially*.” (Primary Care)
**Individual—innovation deliverers (providers)**	Provider buy‐in: “*Provider buy‐in and education is an anticipated clinic barrier. Some primary care clinicians are proponents of long‐acting injectables for treatment of HIV. However, some providers are reluctant to update regimens for patients, particularly those patients that are virally suppressed*.” (Academic Medical Centre)
	Concerns about resistance: “*The cabotegravir ‘tail’ is certainly concerning for our patients who become lost to follow‐up. We worry about our patients developing resistance to the integrase inhibitor class and are trying to figure out ways to best educate patients on this issue*.” (Academic Medical Centre)
	Provider comfort: “*As this is a new therapy and there is a new mechanism of delivery not all providers in our practice currently feel comfortable with the process of initiating someone on LAI ART [long acting injectable antiretroviral treatments]. Having the staff to create both educational materials but also a consistent workflow and guidance document will help us scale up to more vulnerable patients and help prescribers become comfortable with the process of switching to LAIs*.” (Academic Medical Centre)

At the outer setting, clinics described significant barriers related to financing (*n* = 27/38, 71%). For patients with private insurance, these barriers included denial of coverage, and time‐intensive requirement for obtaining prior authorization. Although iCAB/RPV is covered by the AIDS Drug Assistance Program (ADAP) in some states, others do not currently include it on their formulary. Income thresholds for Medicaid, especially in non‐expansion states, leave some patients uninsured. There are also challenges associated with coverage of the medication under pharmacy versus medical benefits. Some clinics reported that they are only able to procure the medication under one mechanism and not the other, thereby systematically preventing some patients from accessing iCAB/RPV.

At the inner setting, clinics reported multiple logistical barriers, with the most common being infrastructure technology needs, specifically the need for new systems to track, schedule, and coordinate injection visits and drug procurement (*n* = 19, 50%). Given the high administrative burden, clinics also reported work infrastructure and compatibility needs, including the need to both develop new workflows and increase staffing to carry out new workflows related to procuring and delivering iCAB/RPV (*n* = 16, 42%). They also reported physical infrastructure needs, specifically refrigerated space to store the medication and private clinic space to administer the injections (*n* = 4, 11%). Finally, clinics also recognized that to deliver iCAB/RPV equitably, new systems that centred recipient (patient) needs would be necessary, most notably transportation support (particularly for clinics in rural areas without public transportation) (*n* = 13, 34%) and extended clinic hours (*n* = 3, 8%) to account for the more frequent visits required by iCAB/RPV.

Finally, at the individual level, some clinics reported needing to build both innovation recipient (patient) and innovation deliverer (clinical and non‐clinical provider) trust and knowledge about this new treatment option to increase capability and motivation to receive and deliver iCAB/RPV. For example, a third of clinics (*n* = 12, 32%) reported that patients had limited knowledge about injectable treatment and some patients were sceptical or distrusting of the new modality. About a third of clinics (*n* = 11, 29%) also wrote about the need to engage providers at all levels to gain buy‐in and increase technical knowledge and skills to support the equitable delivery of this new, more expensive and logistically challenging‐to‐administer medication, recognizing and centring innovation deliverer needs. Provider concerns and training needs spanned from the practical (e.g. how to integrate iCAB/RPV into clinic workflow) to the technical (e.g. how to inject iCAB/RPV across diverse body types) to clinical concerns (e.g. potential for treatment failure and/or resistance, which patients are eligible/should be prioritized for this treatment).

### 3.6 Clinic potential to use LAI ART as a tool to advance health equity

Evidence of clinics’ culture of human‐equality centredness was most present in clinics’ self‐descriptions and mission statements, some of which explicitly mentioned a commitment to equity or addressing health disparities (*n* = 7, 18%), while more mentioned providing inclusive, holistic, compassionate, culturally appropriate, stigma‐free and affirming care (*n* = 14, 37%). Nearly, all clinics described a commitment to serving patients with disadvantage and/or multiple complex health or social needs (*n* = 31, 82%).

A culture of recipient‐centredness was evidenced by about half of the clinics reporting providing comprehensive primary care (*n* = 22, 58%) or comprehensive sexual health services (*n* = 18, 47%). A limited number of clinics additionally reported providing wraparound services to address social and structural barriers to HIV care which could also potentially be leveraged to deliver iCAB/RPV equitably, including transportation assistance (*n* = 24, 63%); free, sliding scale or financial assistance for care (*n* = 9, 24%); assistance with insurance enrolment (*n* = 5, 13%); intensive outreach or case management (*n* = 5, 13%); housing assistance (*n* = 9, 24%); food assistance (*n* = 17, 45%); mobile or telehealth services (*n* = 7, 18%); and extended or weekend hours (*n* = 6, 16%).

Clinics varied in terms of which populations they prioritized for iCAB/RPV delivery. Clinics prioritized delivering iCAB/RPV to their most marginalized patients struggling to maintain or achieve viral suppression, with the most frequently prioritized patients being patients who identified as Black or Latinx (*n* = 13, 34%) and/or LGBTQ or gender non‐conforming (*n* = 11, 29%), and patients with pill fatigue or pill aversions (*n* = 10, 26%) (Table 1). However, clinics also acknowledged the tension between wanting to prioritize patients unable to become virally suppressed on oral medications with the awareness that current FDA approval is only for virally suppressed patients. Some clinics (*n* = 4, 11%) were open about wanting to start with the most “ideal” or “easiest” patients first as they worked out the details of their programmes and thus prioritized patients with a history of on‐time visits and adherence to oral ART, raising the risk that introduction of iCAB/RPV may exacerbate existing health disparities initially. For example, an AMC shared: *“we currently offer LAI ARV to patients who are currently suppressed on HIV therapy without any evidence of resistance to the NNRTI or integrase inhibitor class. As we scale up, we plan to prioritize patients who are at risk for medication non‐adherence as we feel this is a population who will benefit from LAI.”*

While some clinics reported that they would prioritize patients with adherence challenges (*n* = 8, 21%), no clinics explicitly stated that they would initiate patients who were currently not virally suppressed. Many clinics walked a middle ground, describing LAI ART as a “carrot” that could motivate patients with adherence challenges to adhere to oral ART as a bridge therapy. In a similar vein, some clinics were interested in introducing iCAB/RPV to patients who were newly diagnosed (*n* = 3, 8%) and spoke about coupling injectable treatment with immediate or same‐day ART strategies and using oral ART as a bridge therapy for only as long as was necessary to achieve viral suppression.

### 3.7 Resources needed to support iCAB/RPV implementation

Given these challenges, a large percentage of clinics reported needing technical assistance to develop protocols and more efficient workflows (*n* = 18, 47%), specifically, strategies to address challenges associated with obtaining coverage for the cost of medication (*n* = 8, 21%). Many clinics described struggling with workflows that were labour‐ and time‐intensive and, therefore, not scalable. Clinics sought more streamlined protocols for all aspects of iCAB/RPV delivery and workflows that have been proven effective and reflect best practices. In addition to top‐down guidance, many clinics were eager for opportunities to learn from other implementing clinics to identify best practices and strategies for overcoming implementation challenges (*n* = 12, 32%).

Clinics also discussed the need for dedicated staff to provide care coordination and benefits navigation as well as ensure that patients had the wraparound support they needed (e.g. transportation assistance) to attend injection visits on time (*n* = 17, 45%). Many clinics spoke about potential task‐shifting strategies, including enlisting student pharmacists and student nurses to assist with prior authorizations, medication shipment coordination and scheduling; peer navigators to assist with insurance enrolment, prior authorizations and appointment reminders; and HIV case managers to identify and provide wraparound services and provide initial education.

Clinics reported a need for patient‐facing education and marketing materials to increase awareness about iCAB/RPV and increase demand (*n* = 7, 18%) as well as support engaging communities around this new treatment (*n* = 6, 16%). Additionally, clinics also noted the need for provider education, training and engagement, not only to increase provider knowledge about both the practical as well as clinical aspects of delivering iCAB/RPV but also to increase provider buy‐in that CAB/RPV could be a good treatment choice for some patients.

## 4 DISCUSSION

The ALAI UP request for applications yielded 38 submissions from diverse clinical sites across the United States, highlighting not only widespread interest in offering iCAB/RPV but also the need for implementation support to help clinics introduce and scale up their programmes equitably. Clinics at all stages of implementation applied to participate in ALAI UP, with the most applications from clinics that were just starting to implement iCAB/RPV. However, applications from early adopting clinics where more than 25 patients have been initiated on iCAB/RPV indicated that even these clinics now face challenges scaling up their iCAB/RPV programmes and require additional technical assistance and support to address entrenched barriers and develop more equitable and efficient workflows and protocols. We contribute to the burgeoning literature around iCAB/RPV implementation by using the CFIR to describe barriers to iCAB/RPV introduction and scale up, potential for equitable implementation, as well as needed supports as reported by diverse clinics from across the United States delivering (or trying to deliver) iCAB/RPV to their patients in a non‐research context. Our analysis revealed three core findings, demonstrating the interconnectedness of the different CFIR domains and highlighting areas for future research. Findings also informed the development of a multicomponent implementation support package to facilitate the equitable implementation of iCAB/RPV in eight selected ALAI UP sites, components of which we describe below and will be made publicly available at www.alai‐up.org in the future.

Our first core finding was that across all ALAI UP applicants, the most significant barrier to iCAB/RPV implementation is the high cost of the medication (innovation domain), which interacts with inconsistencies in coverage by individual private insurance companies and local Medicaid/ADAP context (outer setting domain), and clinics’ own capabilities (e.g. staffing, infrastructure and financial resources; inner setting domain) to manage these challenges. Innovation cost was the barrier raised by the largest number of clinics (71%), which reported significant challenges covering the high cost of iCAB/RPV for their patients. Cost issues ranged from challenges with private insurance denials, restrictions or onerous prior authorization requirements, to challenges with coverage in states without Medicaid expansion and/or states that had not authorized iCAB/RPV on their Ryan White HIV/AIDS Program ADAP formulary. In addition to the high cost of the medication itself, costs associated with injection administration can be a barrier for clinical sites and patients. Inconsistencies related to iCAB/RPV coverage as a pharmacy benefit (which is pre‐paid by insurance) or a medical benefit (which must be pre‐paid by the clinic and recouped after administration) is another significant barrier to implementation. In our sample, only large academic medical centres and hospitals had the financial resources and administrative processes in place to provide iCAB/RPV to patients with medical benefits. Together, these coverage challenges have resulted in highly inconsistent access to iCAB/RPV, potentially exacerbating health disparities and severely limiting the public health impact of this new treatment option.

Despite the significant challenge presented by uneven coverage for iCAB/RPV, only a few studies have directly explored or addressed this issue [12, 15, 18, 19]. Most implementation studies to date were conducted prior to FDA approval of iCAB/RPV when the cost of iCAB/RPV had not yet been announced. During this period, coverage challenges were still theoretical [3, 14, 15, 20, 21] and the medication was made available to clinics and patients in a research context [12, 13, 14]. As a result, few resources or strategies exist to help clinics navigate coverage issues.

To support sites in addressing these challenges, ALAI UP is developing and providing clinics with job aids and coverage tracking tools, technical assistance in how to use these tools, and facilitating opportunities for sites to share best practices for navigating and obtaining coverage for iCAB/RPV through communities of practice. An area of future research is the extent to which inner setting facilitators (e.g. job aids, technical assistance) can overcome the significant outer setting barrier of coverage or whether structural‐level implementation strategies at the outer context are a pre‐requisite [1, 22]. For example, pressure on drug manufacturers to bring down the cost of iCAB/RPV in addition to legislative action mandating coverage by insurance companies (similar to the mandatory coverage of PrEP medication, including injectable cabotegravir, under the Affordable Care Act, and inclusion of injectable cabotegravir in the US Preventive Services Task Force list of recommended interventions) may be necessary conditions to overcome implementation challenges [23].

Our second core finding was that clinics are motivated to provide injectable cabotegravir/rilpilvirine to populations who have historically struggled to achieve or sustain viral suppression with oral ART (inner setting domain), and many have comprehensive health and wraparound services that could support expanded use, but clinics are reluctant to do so given the current FDA label and available clinical trial evidence that evaluated iCAB/RPV use only in patients who were virally suppressed (innovation domain) leading to prescribing practices that may exacerbate health disparities [10]. At the time of application, clinics were most comfortable talking about strategies to use oral ART as a bridge to help patients who recently acquired HIV and patients currently virally unsuppressed achieve viral suppression before transitioning to iCAB/RPV.

As clinics gain experience with providing iCAB/RPV to a wider range of patients with more diverse treatment, visit attendance, and viral suppression histories, and as evidence from Ward 86, other demonstration projects, and compassionate use case studies examining the use of iCAB/RPV in patients with unsuppressed viral load continues to grow, trends in which patients are prioritized for and/or deemed good candidates for iCAB/RPV may change in the near future [9, 10, 11, 24, 25, 26]. Additionally, there have been calls for alternative study designs to expand the evidence for offering iCAB/RPV and other future LAI ARTs to people living with HIV with viraemia [10].

To support efforts to expand access to iCAB/RPV within ALAI UP, we have created a standardized reporting infrastructure for routinely collecting patient‐level data from across ALAI UP clinics delivering iCAB/RPV with the goal of collecting clinical data that are “fit for use” to evaluate key clinical outcomes for iCAB/RPV, including initiation, on‐time injections, viral suppression and discontinuation across diverse populations under real‐world conditions. Such real‐world data from ALAI UP and other initiatives to monitor iCAB/RPV outcomes (e.g. registries) can potentially be used to support an extension of the iCAB/RPV FDA label beyond maintenance therapy to include persons with HIV who are not yet virally suppressed through programmes such as the FDA's pilot Advancing Real World Evidence Program. Access to and eligibility for iCAB/RPV could also be expanded through strategies that support patients with viraemia achieve viral suppression with oral ART as a bridge therapy before transitioning to iCAB/RPV. For example, ALAI UP is providing clinics with training in Motivational Interviewing (MI), which is an evidence‐informed practice that elicits and reinforces patients’ own arguments for behaviour change [27]. Clinics can use MI to support patients to achieve viral suppression with oral ART and sustain injection visit schedules. Similarly, the LATITUDE study is examining the use of iCAB/RPV in patients with a history of viraemia and is providing enhanced oral ART adherence support including financial incentives to help patients with viraemia achieve viral suppression before initiating iCAB/RPV [28]. Together, these strategies may increase the likelihood that iCAB/RPV can operate as a tool to enhance health equity and transform HIV treatment options into treatment choice.

Our third core finding is that clinics varied considerably when estimating their projected programme reach, with estimates ranging from 1% up to 65%, reflecting differences in how clinics see iCAB/RPV playing a role within their larger HIV treatment programmes as well as differing perceptions and experiences with barriers to iCAB/RPV implementation across CFIR domains. Some clinics were reluctant to set explicit goals for the number of patients they intended to initiate on iCAB/RPV because of the (justified) belief that iCAB/RPV is not a superior therapy to existing oral ART (for patients who were consistently suppressed on oral ART; innovation domain). In addition, how many patients initiated iCAB/RPV was a matter of patient choice (inner setting domain) and thus not a metric that should be improved upon by clinics. Other clinics found it challenging to set goals given the uncertainty around whether, when and how certain implementation barriers would be overcome.

Goal setting is nonetheless critical for monitoring, evaluating and improving a programme's effectiveness and ensuring that scale‐up and delivery of iCAB/RPV is equitable [29]. Goals should be person‐centred and tied to clinics’ programme implementation goals and objectives. Goals should also inform the development and routine refinement of clinic protocols and workflows. To support the more equitable delivery of iCAB/RPV, clinics selected by ALAI UP will receive protocol templates (as well as technical assistance to support the use and implementation of these protocols) that guide clinics through questions that consider the equity implications of each protocol and workflow decision. For example, a protocol template addressing patient education asks sites to consider the equity implications of educating all patients (eligible or not) about iCAB/RPV versus educating only those patients considered “ideal candidates.” ALAI UP clinics will also receive support in programme monitoring, evaluation and learning, which will entail a regular facilitated examination of the process and clinical outcome data to evaluate the extent to which existing protocols, workflows and implementation strategies are centring equity and achieving their intended purpose and helping clinics meet programme goals across all clinic patients as well as specifically within priority populations. When goals are not met, ALAI UP sites will be supported in learning from these data and modifying clinic procedures as necessary.

Our secondary analysis of ALAI UP application data has several limitations. First, results may be subject to sampling bias, as perspectives included in this analysis may only represent those clinics that were aware of the ALAI UP opportunity, were interested in equity‐centred iCAB/RPV implementation support and had the capacity to submit an application in time to be considered. Second, as the primary source of data for this analysis were responses to prescribed questions in an application, our findings may be subject to social desirability bias, as responses may be biased towards answers that clinics believed would increase their likelihood of being selected for ALAI UP and may not reflect the full experience of clinics. We also did not collect information on the role of the person or people who completed the questionnaire on behalf of the clinic (e.g. medical director, administration) and, therefore, responses may only reflect some clinic perspectives. Third, social determinants of health variables (e.g. food insecurity, housing instability, financial strain) were not defined in the application; therefore, how clinics interpreted, defined and reported these variables could vary, leading to measurement bias. Finally, fourth, we did not collect information on clinic participation in iCAB/RPV clinical trials (though no clinics reported clinical trial participation). Additionally, given the relatively small sample size, it was not possible to conduct a formal analysis by geography, clinic type or other dimension; however, many clinics shared similar barriers to implementation despite differences in geography, patients served, context and implementation stage. Future studies could consider examining how implementation barriers, facilitators and processes may vary by these dimensions.

## 5 CONCLUSIONS

This account provides one of the first looks at real‐world implementation challenges and needs across diverse clinics across the United States striving to implement iCAB/RPV equitably 2 years after iCAB/RPV was first approved by the FDA. Despite differences in geography, patients served, context and implementation stage, many clinics shared common implementation barriers at the individual, inner and outer contexts, presenting an opportunity for common solutions and shared learning that can accelerate the implementation of iCAB/RPV. However, for iCAB/RPV to be a tool to help End the HIV Epidemic, dedicated resources centred on equity and relevant to context and population are needed to help deliver iCAB/RPV to patients not currently being well served by available oral ART [1, 30]. Without this support, the introduction of LAI ART risks exacerbating, not ameliorating, health disparities.

## COMPETING INTERESTS

The authors have no competing interests to declare.

## AUTHORS’ CONTRIBUTIONS

NN, BL, SG and KM conceptualized and wrote the paper. NN, BL, SG, CC, JZ, KK, MT, JB, CC, AM, DC, BK and KM analysed the data. All authors have read and approved the final manuscript.

## FUNDING

This programme is supported by the Health Resources and Services Administration (HRSA) and the Minority HIV/AIDS Fund of the US Department of Health and Human Services (HHS). The contents are those of the authors and do not necessarily represent the official views of, nor an endorsement, by HRSA, HHS or the US Government.

## CME STATEMENT

This article is published as part of a supplement supported by unrestricted educational grant by ViiV Healthcare.

Credits Available for this Activity: American Medical Association (AMA Credit).

Washington University School of Medicine in St. Louis designates this enduring material for a maximum of 1 AMA PRA Category 1 Credit™. Physicians should claim only the credit commensurate with the extent of their participation in the activity.

## Supporting information


**Supporting file 1**: ALAI UP Project Clinical Site Questionnaire

## Data Availability

The data that support the findings of this study are available on request from the corresponding author. The data are not publicly available due to privacy restrictions.
