# Supplementary material for: Long‐acting injectable ART to advance health equity: a descriptive analysis of US clinic perspectives on barriers, needed support and programme goals for implementation from applications to the ALAI UP Project
Source: J Int AIDS Soc. 2024 Jul 5;27(Suppl 1):e26282. doi: 10.1002/jia2.26282 (PMC11224578; doi:10.1002/jia2.26282)
Supplement: Supplementary file 1 — Supporting file 1: ALAI UP Project Clinical Site Questionnaire [file JIA2-27-e26282-s001.pdf]

ALAI UP Project Clinical Site Questionnaire

Instructions   Please note that you must submit your answers to this questionnaire on the online application platform (REDCap).   We recommend that you save your answers in the Word document before copying them into REDCap.   If you are not yet ready to submit your application, you can scroll to the bottom of the page and click the button 'Save and Return Later'.   To return to your application, after clicking "Save and Return Later' you can do one of three things: 1) Enter an email address for a direct link to return to your application. This does not have to be the original email address used when requesting REDCap access.   2) Bookmark the 'Saved Response Page'.   3) Go to https://redcap.sac-cu.org/surveys/ and enter your site's unique application code (same process as the first time you logged in.   When your application is ready to submit, click the 'Submit' button.

What is the name of your clinic? __________________________________

What is the name of the person submitting this questionnaire? __________________________________

What is the email address of the person submitting the questionnaire? __________________________________

Introduction to your clinic

1. Please tell us a little bit about your clinic, specifically: (2-5 sentences)   Your clinic's mission   Geographic area served   Populations served   Main funding source (e.g., Ryan White-funded)   Anything you think is important for us to know ____

2. To give us a sense of the needs of people with HIV who access services at your clinic, please select the descriptor that best describes approximately how many of your patients with HIV experience the factors below.

	None	A few	About half	Most	All
Food insecurity	○	○	○	○	○
Housing instability	○	○	○	○	○
Patients best served in a language other than English	○	○	○	○	○
Financial strain	○	○	○	○	○
Lack of transportation/access to public transportation	○	○	○	○	○
Any other social or structural determinant of health that acts as a barrier to care for your patient population (please specify)	○	○	○	○	○

Please specify the social or structural determinant of health that acts as a barrier to care for your patient population. __________________________________

Experience with implementation of HIV treatment

3. Please describe HIV care and wrap around services offered at your clinic. (2-5 sentences) __________________________________

Access to care is negatively impacted by social and structural determinants of health (e.g., racism, heterosexism, poverty, transportation, housing instability).

4. Please share any examples of how your clinic has engaged with the local community to understand patient needs as they relate to social and structural determinants of health. (2-5 sentences) __________________________________

5. Please share any examples of how your clinic has addressed social or structural barriers when delivering HIV care. (2-5 sentences) __________________________________

6. How might lessons learned from community engagement and/or addressing social or structural barriers to HIV care inform how you deliver (or plan to deliver) long-acting cabotegravir/rilpivirine? (2-5 sentences) __________________________________

7. Clinics at all stages of implementing long-acting cabotegravir/rilpivirine will be considered for participation in the ALAI UP Project. Please select the option below that best describes the stage of implementation at your clinic.

- ○ We are just starting to consider whether implementation of long-acting cabotegravir/rilpivirine is possible in our setting.
- ○ We have just started implementing long-acting cabotegravir/rilpivirine and have fewer than 25 patients currently on the injectable regimen.
- ○ Long-acting cabotegravir/rilpivirine implementation has been fully integrated into services for people with HIV.

8. Approximately when did you initiate your first patient on long-acting cabotegravir/rilpivirine? MM/YY ____________________________

9. Approximately how many people with HIV do you currently have on long-acting cabotegravir/rilpivirine? ____________________________

10. What proportion of your patients with HIV do you hope to initiate on long-acting cabotegravir/rilpivirine within the first year of implementation? (0-100%) ____________________________

Motivation for applying to The ALAI UP Project

11. Why are you interested in participating in The ALAI UP Project? (2-5 sentences) __________________________________

12. Please describe the strengths your clinic brings to implementing long-acting cabotegravir/rilpivirine. (2-5 sentences) __________________________________

13. Please list three anticipated or experienced barriers to implementation of long-acting cabotegravir/rilpivirine at your clinic. (2-5 sentences) ______________________

14. What patient populations are your top priority/priorities for long-acting cabotegravir/rilpivirine implementation and why? (1-3 sentences) ______________________

15. What type of resources could ALAI UP provide that would enable you to address these barriers and best reach these priority patients? (2-5 sentences) ______________________

Organizational Structure and Staffing Plan

16. Having an identified Project Champion at a clinical site is associated with success in implementation. Do you have a person who will serve as the Project Champion?
- ◯ Yes
- ◯ No

17. Please describe the Project Champion's role in the clinic, how long they have been at the clinic, and any experience championing a new initiative in a clinical setting. (4-6 sentences) ______________________

18. How will you identify a Project Champion or create/modify a role accordingly? (4-6 sentences) ______________________

19. Please describe how the Project Champion will strive to get clinical and administrative buy in for the introduction of long-acting cabotegravir/rilpivirine? (1 to 3 sentences) ______________________

20. So that we can better understand your organizational structure and staffing capacity, please describe the responsibilities and number of staff in each role that are involved in HIV-related care.

Do you have a nurse practitioner (NP) or doctor (MD) at your clinic who is involved in HIV-related care?
- ◯ Yes
- ◯ No

Please describe the responsibilities of the NP / MD involved in HIV-related care. ______________________

How many NPs / MDs at your clinic are involved in HIV-related care? ______________________

Do you have a registered nurse (RN) at your clinic who is involved in HIV-related care?
- ◯ Yes
- ◯ No

Please describe the responsibilities of the RN involved in HIV-related care. ______________________

How many RNs at your clinic are involved in HIV-related care? ______________________

Do you have a social worker at your clinic who is involved in HIV-related care?	◯ Yes<br>◯ No
Please describe the responsibilities of the social worker involved in HIV-related care.	______________________
How many social workers at your clinic are involved in HIV-related care?	__________________
Do you have a benefits coordinator at your clinic who is involved in HIV-related care?	◯ Yes<br>◯ No
Please describe the responsibilities of the benefits coordinator involved in HIV-related care.	______________________
How many benefits coordinators at your clinic are involved in HIV-related care?	__________________
Do you have a peer navigator at your clinic who is involved in HIV-related care?	◯ Yes<br>◯ No
Please describe the responsibilities of the peer navigator involved in HIV-related care.	______________________
How many peer navigator at your clinic are involved in HIV-related care?	__________________
Are there other staff at your clinic not yet listed who are involved in HIV-related care?	◯ Yes<br>◯ No
What is their role? What are their responsibilities? How many people do you have in this role at your clinic?	______________________

21. The ALAI UP Project will work with your clinic's Implementation Team to co-develop protocols and workflows to support implementation of long-acting cabotegravir/rilpivirine. These protocols and workflows will include benefits navigation, clinical services, psychosocial support, and logistics and scheduling.

Please describe who (role and names) will be responsible for working with the ALAI UP Project Team as the Clinical Site Implementation Team to co-develop protocols in the areas listed below. The same individual may be listed multiple times, however the Implementation Team should include at least three different individuals or an explanation of an alternative implementation approach if that is not possible.

Who will be responsible for patient education & decision-making counseling? Role Name (if known)	______________________
Who will be responsible for determination of benefits coverage / payor source? Role Name (if known)	______________________
Who will be responsible for drug procurement, storage, delivery ? Role Name (if known)	______________________

Who will be responsible for scheduling visits & tracking patient flow?   Role   Name (if known)

____

Who will be responsible for administration of injections?   Role   Name (if known)

____

Who will be responsible for data reporting?   Role   Name (if known)

____

22. The ALAI UP Project is designed to provide technical and financial support for 3 years. Please describe a sustainability plan that will allow your clinic to sustain full participation in ALAI UP in the event of changing leadership, staff, or clinic priorities. (2-5 sentences)

____

Site Experience and Capabilities to Manage and Report Data for Project Evaluation Purposes

A key goal of The ALAI UP Project is to promote the equitable implementation of LAI ARV through continuous monitoring of implementation process and patient outcomes so that adjustments can be made when necessary. This requires a system that can support data collection as well as data extraction.

23. Does your clinic currently have an Electronic Health Record (EHR) system in use for patient care?

◯ Yes, in use for over 12 months
◯ Yes, in use for less than 12 months
◯ No

24. Please list key HIV indicators that you currently collect to monitor the quality of your HIV services.

____

25. What systems do you have in place that can support collecting and reporting aggregate patient-level data for this project, including data comparing patients on long-acting cabotegravir/rilpivirine to your overall HIV patient population? (1-3 sentences)

____

26. Please describe any internal continuous quality monitoring process at your clinic. (2 to 5 sentences)

____

27. Please describe a recent quality improvement effort that your clinic has undertaken and the result of that effort. (3-5 sentences)

____
